# Detection of Cathelicidin-1 in the Milk as an Early Indicator of Mastitis in Ewes

**DOI:** 10.3390/pathogens8040270

**Published:** 2019-11-28

**Authors:** Angeliki I. Katsafadou, George Th. Tsangaris, Natalia G.C. Vasileiou, Katerina S. Ioannidi, Athanasios K. Anagnostopoulos, Charalambos Billinis, Ilektra A. Fragkou, Elias Papadopoulos, Vasia S. Mavrogianni, Charalambia K. Michael, M. Filippa Addis, George C. Fthenakis

**Affiliations:** 1 Veterinary Faculty, University of Thessaly, 43100 Karditsa, Greece; 2 Proteomics Research Unit, Biomedical Research Foundation of Academy of Athens, 11527 Athens, Greece; 3 Laboratory of Parasitology and Parasitic Diseases, Faculty of Veterinary Medicine, Aristotle University of Thessaloniki, 54124 Thessaloniki, Greece; 4 Dipartimento di Medicina Veterinaria, Università degli Studi di Milano, Via G. Celoria 10, 20133 Milan, Italy

**Keywords:** biomarker, cathelicidin-1, diagnosis, mastitis, proteomics, sheep, somatic cell counts

## Abstract

The objective of the study was the investigation of the behaviour of cathelicidin-1 in the milk after experimental infection with two prominent bacterial pathogens (experiment 1: *Mannheimia haemolytica*, experiment 2: *M. haemolytica* and *Staphylococcus chromogenes*) as a potential early indicator for diagnosis of mastitis in sheep. In two experiments, after bacterial inoculation into the udder of ewes, bacteriological and cytological examinations of milk samples as well as proteomics examinations [two-dimensional gel electrophoresis analysis (2-DE) and matrix-assisted laser desorption/ionization time-of-flight mass spectrometer (MALDI-TOF MS) analysis] were performed sequentially. Cathelicidin-1 was detected and spot densities obtained from PDQuest v.8.0 were recorded. Associations were calculated between cell content and spot densities as well as between presence of mastitis in a mammary gland at a given time-point and detection of cathelicidin-1 in the respective milk sample. All inoculated mammary glands developed mastitis, confirmed by the consistent bacterial isolation from mammary secretion and increased leucocyte content therein. Spot density of cathelicidin-1 in samples from inoculated glands increased 3 h post-inoculation; spot density of cathelicidin-1 in samples from inoculated glands was higher than in samples from uninoculated controls. There was clear evidence of correlation between cell content and cathelicidin-1 spot densities in milk samples. There was significant association between presence of mastitis in the mammary gland and detection of cathelicidin-1 in the respective milk sample; overall accuracy was 0.818—this was significantly greater during the first 24 h post-challenge (0.903) than after the first day (0.704). In conclusion, detection of cathelicidin-1 in milk was significantly associated with presence of mastitis in ewes. The associations were stronger during the first 24 h post-infection than after the first day. Cathelicidin-1 has the advantage that it can be a non-specific biomarker, as simply a “positive”/“negative” assessment would be sufficient.

## 1. Introduction

In sheep flocks, mastitis is an important disease. It is difficult to control and hence has significant financial impact and welfare concerns [1,2,3]. The disease is the consequence of bacterial invasion into the mammary gland through the teat, bacterial multiplication, and a subsequent inflammatory reaction. Various bacteria can cause mastitis in ewes, *Staphylococcus* spp. and *Mannheimia haemolytica* being the most frequent ones [3]. Furthermore, many factors can predispose animals to the disease [4].

Diagnosis of clinical mastitis takes place through the recognition of clinical signs occurring in the affected mammary glands. Diagnosis of subclinical mastitis is achieved through the combination of bacteriological and cytological findings. The early diagnosis of intramammary infection is paramount for its effective treatment [1]. The process of infection and the ensuing damage to the mammary gland are rapid, as histological lesions occur within 24 h of infection [5]. Therefore, early recognition of the disease is important for minimizing mammary gland lesions and restoring health.

Diagnostic methods include clinical examination, imaging techniques (ultrasonographic examination, endoscopy), bacteriological examination of milk samples, immunological tests, identification of biomarkers (cytological examination of milk, measurement of milk electroconductivity) [6]. Each method has limitations that might compromise the speed and the accuracy of diagnosis. Until now, bacteriological and cytological examinations of milk samples have been considered as the most useful techniques for diagnosis of subclinical mastitis. Nevertheless, various novel methods have also been evaluated. Among those, detection of cathelicidin has significant advantages as a mammary gland inflammation biomarker for diagnosing the infection. Previous works have already pointed out the value of cathelicidins for mastitis detection in ewes [7,8].

The objective of the study was to investigate the behaviour of cathelicidin-1 in milk after experimental infection with two prominent bacterial pathogens as a potential early indicator for diagnosis of mastitis in sheep. The use of experimental infection models allowed full control over mastitis development and accurate recognition of the disease.

## 2. Results

### 2.1. Conventional Examinations

In both experiments performed, before inoculation, the udders of all ewes appeared clinically healthy. No bacteria were isolated from any milk sample. California Mastitis Test (CMT) scores were negative. Somatic cell counts were <0.45 × 10^6^ cells mL^−1^. Observation of milk films revealed only scarce presence of macrophages (on average, one cell per 10 fields).

In experiment 1, all ewes developed clinical mastitis after inoculation. Starting 12 h post-inoculation and until day (D) 4, in all ewes, *M. haemolytica* was isolated in pure culture from samples from the inoculated side of the udder (in total, 12 isolations from 22 samples). Cell content was increased therein (in total, 20 of 22 samples had >‘1’ CMT and abundant leucocytes with ≥90% neutrophils).

Moreover, in mammary tissue samples from the inoculated side of the udder, leucocytic infiltration (neutrophils, lymphocytes) was seen histologically. Furthermore, intra-alveolar live and exhausted neutrophils, extravasation, and destruction of epithelial cells and alveoli were evident in some parts of the samples examined (Figure S1); in some ewes, conspicuous haemorrhage was also noted.

In experiment 2, the ewes inoculated with *M. haemolytica* developed clinical mastitis. The ewes inoculated with *Staphylococcus chromogenes* developed subclinical mastitis. Starting on D0 + 3 h, the challenge organism was isolated in pure culture from the inoculated mammary glands in all sampling occasions (in total, 30 isolations from 30 samples). Increased cell content was evident from D0 + 6 h (in total, 22 of 30 samples had ≥‘1’ CMT and ≥0.5 ×10^6^ cells mL^−1^ and abundant leucocytes with ≥90% neutrophils; of these, 11 were from ewes inoculated with *M. haemolytica* and 11 from ewes inoculated with *S. chromogenes*).

In both experiments, no uninoculated side of the udder of the ewes developed clinical or subclinical mastitis (*p* < 0.004 versus the inoculated sides). No bacteria were isolated from any milk sample from these sides, and no increased cell content was recorded (*p* < 0.001 versus the inoculated sides). In experiment 1, no lesions were evident in any tissue sample from the non-inoculated side of the udder of the experimental ewes. In experiment 2, somatic cell counts remained <0.43 × 10^6^ cells mL^−1^ in all milk samples during the study (*p* < 0.001 versus the inoculated sides).

### 2.2. Proteomics Examinations

In both experiments performed, before inoculation, no cathelicidin-1 was detected in any milk sample from any ewe.

**Figure 1:**
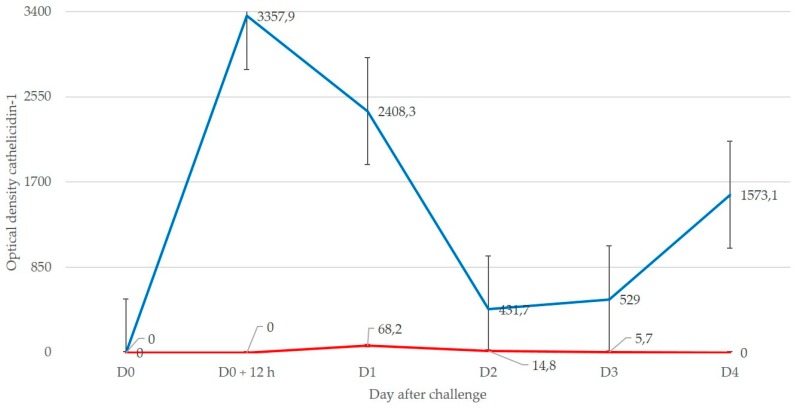
Mean spot densities of cathelicidin-1 in two-dimensional gel electrophoresis analysis (2-DE) gels obtained from sequential milk whey samples from inoculated (blue line) or uninoculated (red line) side of the udder, subsequently to inoculation of one teat with *M. haemolytica* (experiment 1).

In experiment 1, starting on D0 + 12 h, in all ewes, cathelicidin-1 was detected in samples from the inoculated side of udders (in total, in 19 of 22 samples). Starting on D1 and until D3, in three ewes, it was also detected in samples from the uninoculated side of udders (in total, in 5 of 22 samples) (*p* < 0.001 versus inoculated glands). Mean spot densities of cathelicidin-1 in samples from inoculated sides on D0 + 12 h on D1 and on D4 were significantly higher compared to D0 (*p* = 0.05), as were the differences between some sampling points after inoculation (*p* = 0.05). Spot densities in samples from uninoculated sides were not significantly different compared to D0, nor were differences between the sampling points after inoculation (*p* > 0.30). Differences in mean spot densities of cathelicidin-1 in samples from inoculated versus uninoculated side throughout the study were significant: 1671.9 ± 774.4 versus 22.1 ± 14.2 (*p* = 0.005). Mean spot densities in the various sampling points of the study were significantly higher in samples from the inoculated than from the uninoculated side of the udders (*p* = 0.05) (Figure 1 and Figure S2, Table S1).

**Figure 2:**
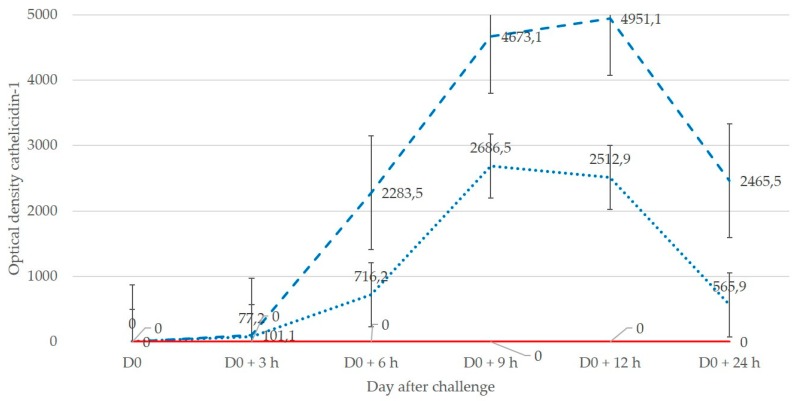
Mean spot densities of cathelicidin-1 in 2-DE gels obtained from sequential milk whey samples from inoculated (blue line: dashed inoculated with *M. haemolytica*, dotted inoculated with *S. chromogenes*) or uninoculated (red line) side of the udder, subsequently to inoculation of one gland with *M. haemolytica* or *S. chromogenes* (experiment 2).

In experiment 2, starting on D0 + 3 h, in all ewes, cathelicidin-1 was detected in samples from the inoculated side of udders (in total, in 29 of 30 samples; of these, 14 were from ewes inoculated with *M. haemolytica* and 15 from ewes inoculated with *S. chromogenes*). It was not detected in any sample from the uninoculated side of udders (*p* < 0.001 versus inoculated glands). Mean spot densities of cathelicidin-1 in samples from inoculated sides on D0 + 6 h and thereafter were significantly higher compared to D0 (*p* = 0.025), as were the differences between some sampling points after inoculation (*p* ≤ 0.05). Differences in mean spot densities of cathelicidin-1 in samples from inoculated versus uninoculated side throughout the study were significant: 2103.3 ± 562.0 versus 0.0 ± 0.0 (*p* < 0.005). Mean spot densities in the various sampling points of the study were significantly higher in samples from the inoculated than from the uninoculated side of the udders (*p* = 0.05) (Figure 2 and Figure S3, Table S1). Mean spot density of cathelicidin-1 from all samples from inoculated glands of ewes challenged with *M. haemolytica* was higher than that from ewes challenged with *S. chromogenes*: 2895.9 ± 973.3 versus 1312.0 ± 360.7 (*p* = 0.34).

### 2.3. Sensitivity/Specificity of Detection of Cathelicidin-1 for Diagnosis of Mastitis

Cathelicidin-1 was identified in milk samples at the same sampling as (experiment 1: 12 h after inoculation) or earlier than (experiment 2: 3 h versus 6 h after inoculation) increase of cell content. The difference between frequencies of detection on D0 + 3 h was significant: 5/6 samples for cathelicidin detection versus 0/6 samples for increased cell content (*p* = 0.008). The difference on D0 + 6 h was not significant: 6/6 samples for cathelicidin detection versus 4/6 samples for increased cell content (*p* = 0.23).

**Figure 3:**
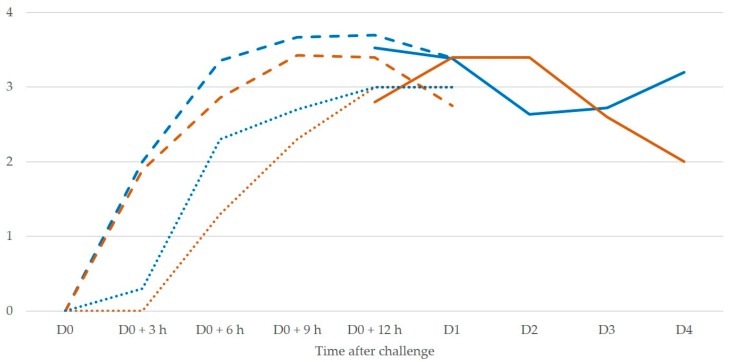
Log_10_ of mean spot densities of cathelicidin-1 in 2-DE gels (blue lines) and mean California Mastitis Test (CMT) scores (brown lines) in sequential milk samples from inoculated side of the udder, subsequently to intramammary infection (solid lines: experiment 1, dashed lines: ewes inoculated with *M. haemolytica* in experiment 2, dotted lines: ewes inoculated with *S. chromogenes* in experiment 2).

There was evidence of correlation between CMT scores and cathelicidin-1 spot densities in milk samples (Figure 3). The correlation coefficient for the cumulative results of both experiments was *r* = 0.398 (*p* < 0.001); the respective values for experiments 1 and 2 were *r* = 0.272 (*p* = 0.023) and *r* = 0.540 (*p* < 0.001). The difference in correlation coefficients between experiments 1 and 2 was significant: *z* = −1.76 (*p* = 0.039). Within experiment 2, the correlation coefficients when results from ewes inoculated with *M. haemolytica* or *S. chromogenes* were taken separately were *r* = 0.604 and 0.704 (*p* < 0.001), respectively. The difference between these two correlation coefficients was not significant: *z* = −0.71 (*p* = 0.24). There was also evidence of correlation between somatic cell counts and cathelicidin-1 spot densities in milk samples in experiment 2. The correlation coefficient was *r* = 0.565 (*p* < 0.001).

Finally, there was significant association between presence of mastitis in a mammary gland on a sampling point and presence of cathelicidin-1 in the respective milk sample (*p* = 6.0 × 10^−7^) (Table 1). Sensitivity and specificity of using presence of cathelicidin-1 for diagnosis of mastitis were 0.912 (95% confidence intervals: 0.763–0.981) and 0.783 (0.684–0.862), respectively. Positive and negative predictive values were 0.608 (0.509–0.698) and 0.960 (0.890–0.986), respectively. Overall accuracy of detection of cathelicidin-1 for diagnosis of mastitis was 0.818 (0.739–0.881).

When analyses were performed separately for the results of each of the two experiments (Table 1), it emerged that there was evidence of significant association between presence of mastitis in a mammary gland and presence of cathelicidin-1 in the respective milk sample in both experiments (*p* = 0.0082 for experiment 1 and *p* = 8.7 × 10^−13^ for experiment 2). In experiment 2, there was also significant association between presence of mastitis in a mammary gland and presence of cathelicidin-1 in the respective milk sample when samples from ewes inoculated with *M. haemolytica* or *S. chromogenes* were considered separately (*p* = 6.1 × 10^−7^ for ewes inoculated with *M. haemolytica* and *P* = 2.3 × 10^−6^ for ewes inoculated with *S. chromogenes*) (Table 1).

Accuracy of detection of cathelicidin-1 for diagnosis of mastitis was 0.704 (0.564–0.820) in experiment 1 and 0.903 (0.810–0.960) in experiment 2. The difference in accuracy between the two experiments was significant (*p* < 0.001). In experiment 2, accuracy of detection of cathelicidin-1 for diagnosis of mastitis in ewes inoculated with *M. haemolytica* was 0.917 (0.775–0.983) and in ewes inoculated with *S. chromogeens* was 0.889 (0.739–0.969). The difference in accuracy between the ewes inoculated with either organism was not significant (*p* = 0.69).

## 3. Discussion

Bacteria can cause damage to mammary epithelial cells as early as 24 h after invasion; hence, mastitis treatment should start as soon as possible [1].

Diagnosis of clinical mastitis is easy based on recognition of clinical signs, which may nevertheless take 24 to 36 h to develop, a period during which bacteria would continue causing damage to the mammary gland epithelium.

For diagnosis of subclinical mastitis, the best method is the combination of bacteriological and cytological examination [6,9]. Nevertheless, there is still a debate regarding thresholds in cytological examination of milk for detection of the inflammatory reaction in the mammary gland of ewes. No well-defined and fully acceptable thresholds of somatic cell counts have been set. Furthermore, a variety of factors unrelated to the infection (e.g., age of ewe, breed of ewe, stage of lactation, number of lactation, milk yield, time of the day at sampling, daily frequency of milking, number of lambs sucking) might influence the somatic cell counts [10], hence affecting diagnosis. These, therefore, should be taken into account when making a diagnosis of subclinical mastitis. Berthelot et al. [11] indicated a valuable diagnostic approach regarding thresholds of cell counts indicating infection. In individual animals, values < 0.5 × 10^6^ cells mL^−1^ indicate a healthy mammary gland, and values > 1.0 × 10^6^ cells mL^−1^ indicate a mammary gland with mastitis, with no need to perform a bacteriological examination of milk samples below or above these thresholds to confirm the problem. In samples with values between 0.5 × 10^6^ and 1.0 × 10^6^ cells mL^−1^, which indicate suspicion of the disease, it is necessary to perform bacteriological examination in milk. Some researchers have defined somatic cell count thresholds to the much lower value of 0.25 × 10^6^ cells mL^−1^ [12] for diagnosing mastitis. All these confirm the lack of consensus regarding thresholds for somatic cell counts to indicate mastitis. In order to improve diagnosis, identification of cell types in milk samples can be performed, given that milk of healthy ewes contains mainly macrophages, whilst during subclinical mastitis, neutrophils (acute phase) or lymphocytes (chronic phase) predominate [5]. This additional criterion has been used extensively in recent studies on ovine mastitis in order to improve the diagnosis [9,13].

In the past, various proteins were evaluated for a potential significance in the diagnosis of mastitis in cows. These included chaperonins, various leucocyte-associated proteins (e.g., cathelicidin, peptidoglycan recognition protein, lymphocyte cytosolic protein 1, macrophage scavenger receptors) and various whey proteins (e.g., *β*-2-microglobulin, *α*-enolase, chitinase-3-like protein 1) [6]. In cows, cathelicidin proteins have been proposed for identification of subclinical mastitis or as indicators of the stage of infection [14,15,16].

In the present study, the potential use of cathelicidin-1 in the diagnosis of mastitis in ewes was evaluated by carrying out experimental infections with two different established mammary pathogens. In both experiments, mastitis was induced, which allowed us to have animals with confirmed disease under fully controlled conditions. From the results of previous experiments [7,17], cathelicidin-1 was singled out for a detailed investigation, particularly during the initial stage of mammary infection. In experiment 2, samples were collected from experimental animals at frequent intervals for 24 h after inoculation. Three ewes were challenged with *M. haemolytica*, whilst another three were challenged with *S. chromogenes*, an organism with confirmed pathogenicity for the mammary gland [5]. This approach was used in order to confirm the validity of cathelicidin-1 as a non-specific biomarker, especially as diagnostic accuracy of using cathelicidin-1 did not differ between ewes inoculated with either pathogen.

In experiment 2, it was observed that 3 h and 6 h after inoculation, cell counts in mammary secretion were between 0.5 × 10^6^ and 1.0 × 10^6^ cells, which would have led to suspicion (not confirmation) of the disease, although the animals already had confirmed mastitis as the result of intramammary inoculation of pathogens. Hence, bacteriological examination would have been necessary to confirm the disease. In contrast, in the same samples, cathelicidin-1 was clearly detected.

This correlation was further reflected in the increased association of presence of cathelicidin-1 in milk with mastitis status. For definition of mastitis, the combination of bacterial isolation and increased somatic cell counts was used. Although in the present study, mastitis was induced experimentally, i.e., the real status of the mammary gland was accurately known, by using this clinical criterion, it has become possible to simulate conditions in the field, where a combination of bacteriological and cytological examination would need to be employed for improved accuracy. It is also noteworthy that when samples were received for proteomics analysis, their precise origin (animal, sampling time-point, inoculated or contralateral gland) was not known by the processing researcher. These details were revealed after processing and appropriate associations were then made. The performance of a blinded experiment further increases the validity of the results.

The greatest advantage of using cathelicidin-1 detection is the early diagnosis of mastitis that may be achieved that way. The comparison of results of correlation analysis in the two experiments shows stronger associations for experiment 2, i.e., when samples were collected in the first 24 h post-infection. In previous studies performed [16,18], samplings started at later time-points, e.g., 24 h post-infection. In contrast, in the present study, samplings started as early as 3 h post-infection, and cathelicidin could be detected in those samples.

Cathelicidin-1 is synthesized in mammary epithelial cells and immediately released upon exposure of these cells to the invading pathogens [19]. Indeed, cathelicidin production occurs before leucocyte influx into the mammary gland. This is in concordance with the present finding of cathelicidin-1 3 h post-infection, at which time-point leucocytes had not yet entered the infected gland. Likely, this is the reason that it provides a more accurate detection of mastitis at an early stage, i.e., before neutrophils would have entered the mammary gland to counteract a bacterial invasion, and thus no increased numbers would be present to indicate abnormal results in somatic cell counting.

Moreover, as cathelicidin-1 is not present in the milk of healthy ewes, there is no need to establish a threshold, hence simply a “positive”/“negative” assessment would be sufficient. On the other hand, this may lead to “false positive” results yielded by presence of cathelicidin-1 in contralateral glands, as indicated in a few samples in the present study.

Moreover, the protein was not found to be associated with specific pathogens. In the present study, it was detected after challenge with either *M. haemolytica* or *S. chromogenes,* and no significant difference was seen in the findings obtained after inoculation with either pathogen. Addis et al. [7] made similar observations upon challenge with *Streptococcus uberis* associated with intramammary infection in ewes. Hence, it can be employed for detection of an infection independently of its causal agent.

Addis et al. [8] described the development of an ELISA test to detect cathelicidins in milk. With further development, a rapid test may be produced for animal-side use. This could be based on that ELISA and could potentially be available for incorporation into milking systems, consequently detecting the protein during machine-milking of animals in a farm. The fact that dairy ewes are routinely milked twice or thrice daily further supports its potential, because it can help in diagnosing an infection quickly. This may be the most beneficial use of detection of cathelicidin-1 as a biomarker for diagnosis of mastitis.

In conclusion, there was an increased correlation of presence of cathelicidin-1 with results of cytological examination. Cathelicidin-1 was detected in milk earlier than increased cell content. Detection of cathelicidin-1 in milk was highly associated with presence of mastitis in ewes. The associations were stronger during the first 24 h post-infection than after the first day. Moreover, cathelicidin-1 has the advantage that it can be a non-specific biomarker, as simply a “positive”/“negative” assessment would be sufficient.

## 4. Materials and Methods

### 4.1. Experimental Overview

Two experiments were performed. In experiment 1, ewes (*n* = 5) were inoculated with *M. haemolytica* strain VSM08L, which was deposited into one teat duct of the ewes. In experiment 2, ewes were inoculated with *M. haemolytica* or *S. chromogenes* strain 6684 (*n* = 3 and 3, respectively) directly into the gland cistern of the udder. Both strains had confirmed pathogenic action for the mammary gland of ewes [20,21]. All challenges were performed on the fifth day post-lambing. The development of mastitis was confirmed by established methods. Milk samples were also collected for proteomics analysis. The principal investigator (AIK), who performed the proteomics analysis, was blinded to the origin of milk samples, (i.e., from inoculated or uninoculated mammary gland or a particular sampling point).

Conditions prescribed by legislation of the European Union in relation to animal experimentation procedures (Council Directive 86/809/EEC) were met during the experiment from which samples were collected. The experiment was carried out under a license for experimental procedures obtained from the Greek Ministry of Agriculture.

### 4.2. Inoculation Procedure

The challenge organisms were grown on Columbia blood agar and checked for purity, then inoculated into soy broth and incubated aerobically at 37 °C for 5 h. Serial dilutions of broth culture into phosphate buffered saline (PBS) (pH 7.3) were performed, and 0.2 mL of the desired dilution was withdrawn and transferred into a sterile plastic syringe for inoculation. 

In experiment 1, ewes were challenged as follows [17,20]. Initially, the teat was disinfected by using iodine povidone solution. Then, a sterile plastic fine catheter 20 G (Abbocath^®^; Abbott, Abbott Park, USA) and 2 mm long was inserted into one teat; the syringe containing the inoculum was attached to the catheter, and the bacterial suspension was deposited into the *ductus papillaris*. The same technique was used to deposit 0.2 mL of sterile PBS into the contralateral teat duct of each ewe as a control. In experiment 2, the ewes were challenged as follows [5,21]. Initially, the teat was disinfected by using iodine povidone solution. Then, a sterile fine plastic catheter 20 G (Abbocath^®^) was inserted into the teat; the syringe containing the inoculum was attached to the catheter, and the bacterial suspension was injected directly into the *sinus lactiferous*. The same technique was used to inject 0.2 mL of sterile PBS into the contralateral mammary cistern of each ewe as a control. In all occasions, after challenge, lambs were kept away from their dams for 2 h.

In experiment 1, each inoculum contained 1200 to 1250 c.f.u. of *M. haemolytica*, as determined by the method of Miles and Misra [22]. In experiment 2, each inoculum contained 50 to 80 c.f.u. of *M. haemolytica* or 1 × 10^6^ to 2 × 10^6^ c.f.u. of *S. chromogenes*, as determined by the method of Miles and Misra [22]. The validity of these challenge models for inducing mastitis has been confirmed in many previous experimental studies [5,20,21,23,24,25,26,27] by using clinical, bacteriological, cytological, ultrasonographic, proteomics and histological methods [6].

### 4.3. Animal Examination, Sampling, Conventional Laboratory Examinations in Samples

In experiment 1, udder examination of ewes was performed immediately prior to challenge (D0) as well as 12 h after challenge (D0 + 12 h) and 1, 2, 3, and 4 days after challenge (D1, D2, D3, D4) (after the D3 sampling, three ewes were withdrawn from the experiment in order to perform a mammary biopsy to obtain a tissue sample that was used for histological confirmation of mastitis, whilst the other two ewes were subjected to mammary biopsy after the D4 sampling [17]). In experiment 2, udder examination was performed immediately prior to challenge (D0) as well as 3, 6, 9, 12 h and 1 d after challenge (D0 + 3 h, D0 + 6 h, D0 + 9 h, D0 + 12 h, D1).

On each of these time-points, a standardized clinical examination of the udder (observation, palpation, comparison between glands) was initially performed, always by the same clinician (author NGCV). The first two squirts of secretion were drawn on the gloved hand of an assisting investigator and assessed [13,20]. All investigators involved in sampling procedures wore disposable, non-sterile latex gloves. The clinician who examined the animals and collected the milk samples changed gloves after procedures in each animal were completed and before moving to the next one.

Then, secretion samples were collected aseptically and separately from each mammary gland (inoculated and uninoculated) of all ewes. The orifice, the edge and the lower half of the body of the teat were disinfected by single-use sterile gauzes, onto which povidone iodine 7.5% (Betadine surgical scrub; Mundipharma Medical Company, Basel, Switzerland) had been poured, followed by wiping off by means of a new sterile gauze; different gauzes were used for each teat. Then, 10 to 15 mL of secretion were collected into a sterile container; separate samples were collected from each mammary gland into separate containers.

Samples were processed for bacteriological examination within 15 min. after collection. Samples were plated onto Columbia 5% sheep blood agar. Media were incubated aerobically at 37 °C for up to 72 h. Bacteria were identified using established techniques [28,29].

The CMT was carried out in milk samples, as described by Schalm et al. [30] for ewes’ milk. The test was performed and always scored by the same person. Five degrees of reaction (“negative”, “trace”, “l”, “2”, “3”) were described [30]. Milk smears were also prepared and air-dried. Leucocyte subpopulations were identified by direct microscopy after staining of milk smears with Giemsa stain. In each case, 100 cells were observed and counted. In experiment 2, the microscopic cell counting reference method was also performed in each milk sample [31,32]. For this, ≥200 cell nuclei were counted in each sample. The number of cell nuclei counted was multiplied by a working factor to provide number of cells per mL of milk [working factor: (20/d) × (100/b)] (d: diameter of microscope field, b: number of stripes counted on slide). Total milk samples examined bacteriologically and cytologically were as follows. In experiment 1, 10 samples were collected from the ewes before inoculation (i.e., on D0), 22 samples were collected from the inoculated side of the udder after experimental infection (i.e., on D0 + 12 h and thereafter), and 22 samples were collected from the contralateral side of the udder on the same occasions (i.e, on D0 + 12 h and thereafter); therefore, in total, 54 samples were evaluated by conventional laboratory examinations. In experiment 2, 12 samples were collected from the ewes before inoculation (i.e., on D0), 30 samples were collected from the inoculated side of the udder after experimental infection (i.e., on D0 + 3 h and thereafter), and 30 samples were collected from the contralateral side of the udder on the same occasions (i.e, on D0 + 3 h and thereafter); therefore, in total, 72 samples were evaluated by conventional laboratory examination. Of these, 6, 15, and 15 were from the ewes inoculated with *M. haemolytica,* and 6, 15, and 15 were from the ewes inoculated with *S chromogenes*.

Mammary tissue samples collected by biopsy were fixed in 10% neutral-buffered formalin and processed by standard histological techniques [17].

### 4.4. Proteomics Analysis

In experiment 1, all samples were processed individually; in total, 54 milk samples were examined (detailed analysis in Section 4.3.). In experiment 2, all samples from inoculated mammary glands were processed individually, but samples from the uninoculated gland of each ewe were mixed and each pooled sample consisted of equal volumes of five individual samples (i.e., those collected 3 h, 6 h, 9 h, 12 h, and 24 h post-inoculation) from the same animal. These were thoroughly mixed, and then a final volume of 10 to 15 mL was taken for processing. In total, 48 milk samples were examined [12 samples collected from the ewes before inoculation (i.e., on D0), 30 samples collected from the inoculated side of the udder after experimental infection (i.e., on D0 + 3 h and thereafter), and 6 samples pooled from samples collected from the contralateral side of the udder on the same occasions (i.e, on D0 + 3 h and thereafter)]. Of these, 6, 15, and 3 were from the ewes inoculated with *M. haemolytica*, and 6, 15, and 3 were from the ewes inoculated with *S chromogenes*.

From each original sample, many Eppendorf tubes were filled with samples of whey for performing duplicate proteomics examinations. All samples were appropriately prepared for proteomics evaluation as described in detail by Katsafadou et al. [17]. Protein content was determined by the Bradford assay [33]. All samples were then assayed by two-dimensional gel electrophoresis analysis (2-DE), as per the detailed method described by Katsafadou et al. [17] and Anagnostopoulos et al. [34].

In experiment 1, image analysis was performed as detailed by Katsafadou et al. [17] and included the entire surface of each gel; spots corresponding to cathelicidin-1 were identified. In experiment 2, image analysis was limited in the region of each gel, where cathelicidin-1 had been located during experiment 1.

Protein identification was performed by peptide mass fingerprinting. Peptide mixtures were analyzed in a MALDI-TOF MS (matrix-assisted laser desorption/ionization time-of-flight mass spectrometer) (Ultraflex, Bruker Daltonics). Matching of peptides and protein searches was carried out in the MASCOT Server 2 (Matrix Science, Boston, USA). The method for protein identification was the same as the one followed by Katsafadou et al. [17]).

Presence of cathelicidin-1 in milk samples collected from individual ewes before or after inoculation was considered. Spot optical densities obtained from PD Quest v.8.0 for each spot of interest on each gel from sample on D0 or after challenge were recorded. In case of multiple spots indicative of the same protein, sums of densities of all spots were taken into account. The spot volume was used as the parametre for quantifying the protein expression.

### 4.5. Mastitis Definition

Mastitis was defined in ewes with (i) clinically evident abnormalities in mammary gland or mammary secretion or (ii) with no clinical abnormalities but in which a bacteriologically positive milk sample with concurrently increased cell content (CMT score ≥‘l’ or cell counts ≥0.5 × 10^6^ cells mL^−1^) plus increased neutrophil and lymphocyte proportion (≥65% of all leucocytes) in Giemsa-stained milk films was detected [6,9].

### 4.6. Statistical Analysis

All data were entered into Excel spreadsheets. Comparisons were made between inoculated and uninoculated sides of the udder in the frequencies of (i) development of mastitis, (ii) isolations of *M. haemolytica* from milk samples, (iii) samples with increased cell content, and (iv) detection of cathelicidin-1 in milk. In pooled samples (experiment 2), in which during the proteomics evaluation, no cathelicidin-1 was detected in any pooled sample, it was assumed that no cathelicidin-1 was present in any of the samples from which each pooled sample was made up. Then, we proceeded with the analysis under this assumption.

A repeated measures mixed effect linear regression model was used to determine whether spot densities changed over the course of the study period. Models were adjusted for repeated measures within animals. The independent variable was the day after challenge. Wilcoxon Signed Rank test was performed to evaluate differences in means of spot densities in samples from inoculated or uninoculated sides of the udder. In experiment 2, the spot densities obtained from inoculated glands of ewes challenged with *M. haemolytica* were compared to those from inoculated glands of ewes challenged with *S. chromogenes* by using the Mann–Whitney test.

Analysis of correlation between CMT scores / somatic cell counts versus spot densities of cathelicidin-1 in milk samples was performed. For calculations, results obtained from each ewe on each sampling point were considered. Correlation coefficients in the two experiments were compared by using the Fisher *r* to *z* transformation. Finally, the association of presence of mastitis in a mammary gland at a given time-point and detection of cathelicidin-1 in the respective milk sample was assessed by using Fisher exact test and taking into account all sampling points in both experiments. Subsequently, accuracy measures (sensitivity, specificity, positive and negative predictive values, overall accuracy) were calculated.

Significance level was set at *p* ≤ 0.05.

## Figures and Tables

**Table:** (a) Experiment 1.

		**Presence of Mastitis**	
		+	-
Presence of cathelicidin-1	+	9	13
	-	3	29

**Table:** (b1) Experiment 2: ewes inoculated with *M. haemolytica*.

		**Presence of Mastitis**	
		+	-
Presence of cathelicidin-1	+	11	3
	-	0	22

**Table:** (b2) Experiment 2: ewes inoculated with *S. chromogenes*.

		**Presence of Mastitis**	
		+	-
Presence of cathelicidin-1	+	11	4
	-	0	21

## Supplementary Materials

The following are available online at https://www.mdpi.com/2076-0817/8/4/270/s1, Figure S1: Histological section of mammary parenchyma, from inoculated side of the udder, with marked intra-alveolar neutrophilic infiltration and destruction of mammary alveoli (experiment 1). Figure S2. 2-DE gels with annotation of cathelicidin-1, obtained from milk samples (whey) collected from the inoculated side of the udder of a ewe before or after inoculation of the ipsilateral teat with *M. haemolytica* (a, b) or from the uninoculated side of the udder of the same ewe (c) (protein identification by MALDI-TOF MS) (experiment 1). Figure S3. 2-DE gels with annotation of cathelicidin-1, obtained from milk samples (whey) collected from the inoculated side of the udder of a ewe before or after inoculation of the ipsilateral gland with *S. chromogenes* (a, b) or from the uninoculated side of the udder (c) (protein identification by MALDI-TOF MS) (experiment 2). Table S1: Spot densities (mean ± standard error of the mean) of cathelicidin-1 in 2-DE gels from sequential milk samples (whey) from inoculated or uninoculated side of the udder, subsequently to bacterial challenge into one side of the udder (protein identification by MALDI-TOF MS).
