# Supplementary material for: Detection of Cathelicidin-1 in the Milk as an Early Indicator of Mastitis in Ewes
Source: Pathogens. 2019 Nov 28;8(4):270. doi: 10.3390/pathogens8040270 (PMC6963440; doi:10.3390/pathogens8040270)
Supplement: Supplementary file 1 [file pathogens-08-00270-s001.pdf]

Supplementary material

Article

Detection of cathelicidin-1 in the milk as an early indicator of mastitis in ewes

Angeliki I. Katsafadou, George Th. Tsangaris, Natalia G.C. Vasileiou, Katerina S. Ioannidi, Athanasios K. Anagnostopoulos, Charalambos Billinis, Ilektra A. Fragkou, Elias Papadopoulos, Vasia S. Mavrogianni, Charalambia K. Michael, M. Filippa Addis and George C. Fthenakis

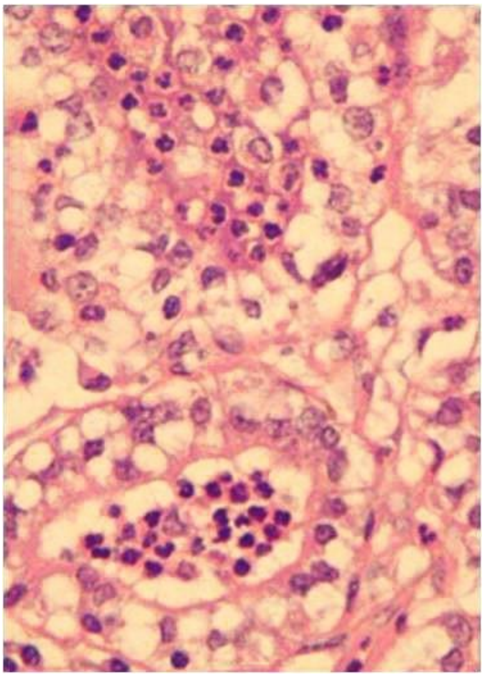

Figure S1. Histological section of mammary parenchyma, from inoculated side of the udder, with marked intra-alveolar neutrophilic infiltration and destruction of mammary alveoli (experiment 1).

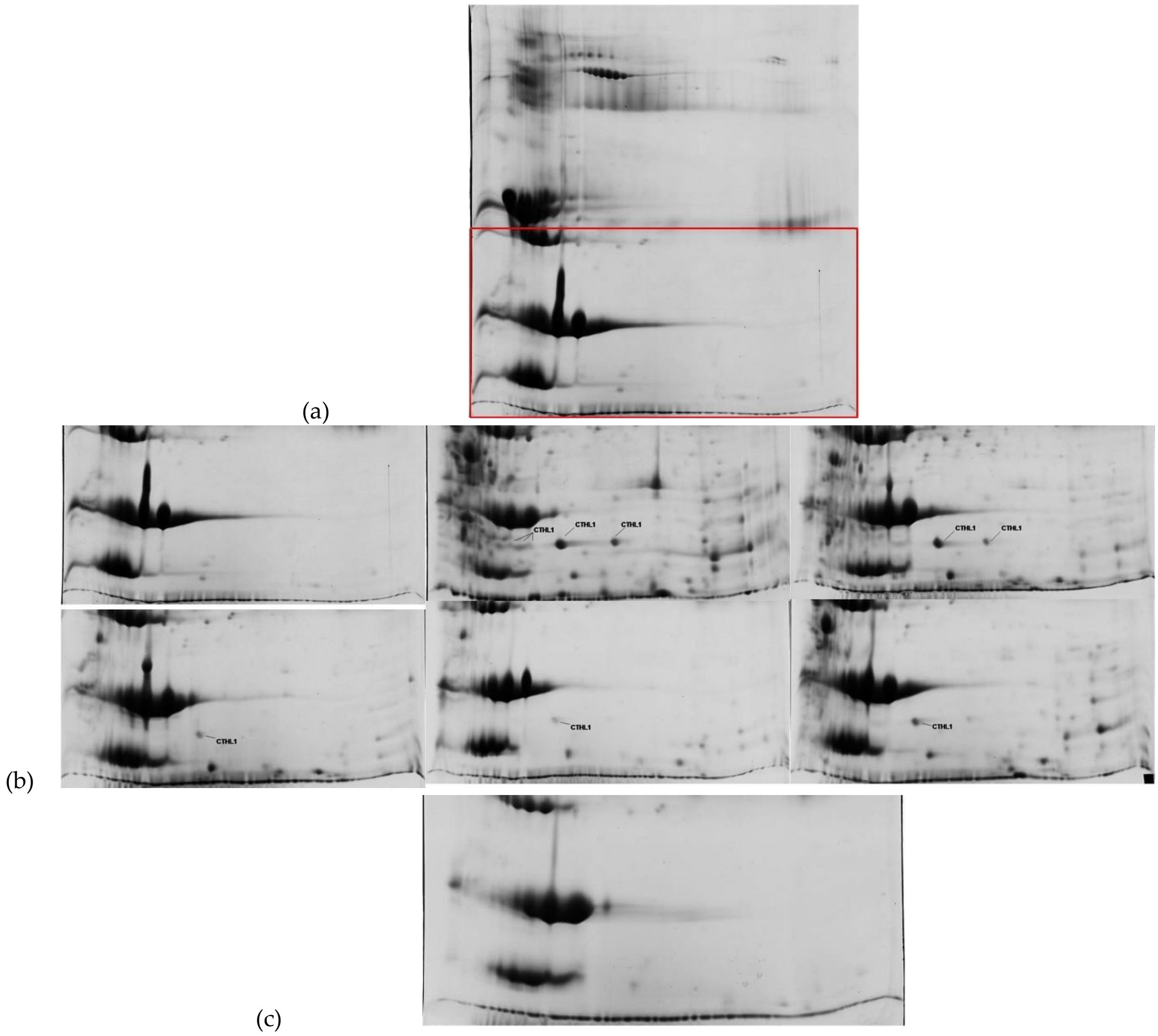

Figure S2. 2-DE gels with annotation of cathelicidin-1, obtained from milk samples (whey) collected from the inoculated side of the udder of a ewe before or after inoculation of the ipsilateral teat with *M. haemolytica* (a, b) or from the uninoculated side of the udder of the same ewe (c) (protein identification by MALDI-TOF MS) (experiment 1).

(a) 2-DE gel obtained from a whey sample before challenge, from the inoculated side of the udder of a ewe; the area in red indicates the region of the gels shown in detail in (b) and (c). (b) Region of 2-DE gels obtained from whey samples before or sequentially after challenge, from the inoculated side of the udder of a ewe; from top left to the right and from bottom right to the right: before inoculation (D0), 12 h after inoculation (D0+12 h), 1 d after inoculation (D1), D2, D3, D4. (c) Region of 2-DE gel obtained from whey sample 12 h after inoculation of the contralateral side of the udder.

Horizontal axis: isoelectric point 3 to 10 (non-linear) from left to right; vertical axis: molecular weight 10 to 100 kDa (non-linear) from bottom to top.

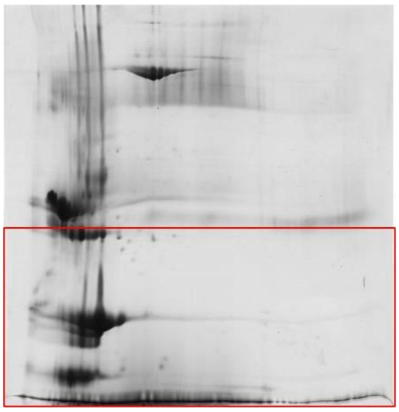

(a)

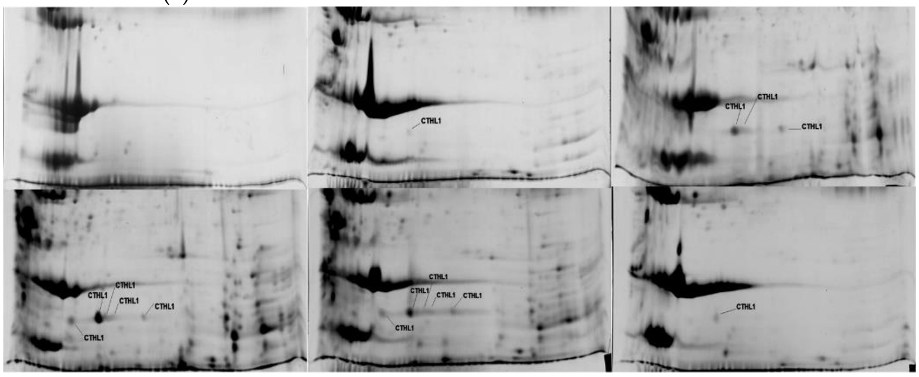

(b)

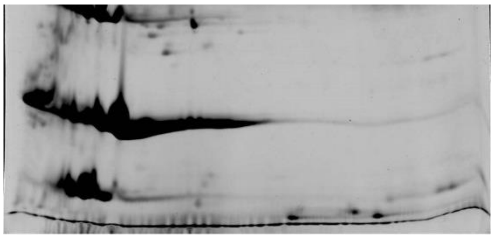

(c)

Figure S3. 2-DE gels with annotation of cathelicidin-1, obtained from milk samples (whey) collected from the inoculated side of the udder of a ewe before or after inoculation of the ipsilateral gland with *S. chromogenes* (a, b) or from the uninoculated side of the udder (c) (protein identification by MALDI-TOF MS) (experiment 2).

(a) 2-DE gel obtained from whey sample before challenge, from the inoculated side of the udder of a ewe; the area in red indicates the region of the gels shown in detail in (b) and (c). (b) Region of 2-DE gels obtained from whey samples before or sequentially after challenge, from the inoculated gland; from top left to the right and from bottom right to the right: before inoculation (D0), 3 h after inoculation, 6 h after inoculation, 9 h after inoculation, 12 h after inoculation, 24 h after inoculation. (c) Region of 2-DE gel obtained from pooled whey samples, from the contralateral to inoculated gland. Horizontal axis: isoelectric point 3 to 10 (non-linear) from left to right; vertical axis: molecular weight 10 to 100 kDa (non-linear) from bottom to top.

Table S1. Spot densities (mean ± standard error of the mean) of cathelicidin-1 in 2-DE gels from sequential milk samples (whey) from inoculated or uninoculated side of the udder, subsequently to bacterial challenge into one side of the udder (protein identification by MALDI-TOF MS).

Udder side	Total spots (n)	Before challenge	After challenge				
			Experiment 1				
		D0	D + 12 h	D1	D2	D3	D4
Inoculated	2.6 ± 0.7[a]	0.0 ± 0.0[x]	3357.9 ± 2687.6[a,k,l,x]	2408.3 ± 2142.0[a,k,m,x]	431.7 ± 278.9[l,m]	529.0 ± 334.8[a,x]	1573.1 ± 1577.8
Un-inoculated	0.8 ± 0.4[a]	0.0 ± 0.0	0.0 ± 0.0[a]	68.2 ± 60.3[a]	14.8 ± 14.7	5.7 ± 5.7[a]	0.0 ± 0.0
			Experiment 2				
		D0	3 h	6 h	9 h	12 h	24 h
Inoculated with any organism	2.3 ± 0.4	0.0 ± 0.0[x]	89.1 ± 25.8	1499.8 ± 721.3[a]	3679.8 ± 1726.0[a,x]	3732.0 ± 1293.2[a,x]	1515.7 ± 1057.2[a,x]
Inoculated with *M. haemolytica*	3.0 ± 0.6	0.0 ± 0.0[x]	101.1 ± 55.5	2283.5 ± 1408.1	4673.1 ± 3661.0[x]	4951.1 ± 2387.6	2465.5 ± 2156.8
Inoculated with *S. chromogenes*	1.7 ± 0.3	0.0 ± 0.0[x]	77.2 ± 10.1	716.2 ± 76.8	2686.5 ± 716.4	2512.9 ± 1085.5	565.9 ± 192.5
Un-inoculated	0.0 ± 0.0	0.0 ± 0.0	0.0 ± 0.0	0.0 ± 0.0[a]	0.0 ± 0.0[a]	0.0 ± 0.0[a]	0.0 ± 0.0

Within each experiment: a = $p \leq 0.05$ between inoculated and non-inoculated glands, k-m = $p \leq 0.05$ between inoculated glands and x = $p \leq 0.05$ for inoculated glands compared to D0.

© 2019 by the authors. Published under the terms and conditions of the Creative Commons Attribution (CC BY) license (http://creativecommons.org/licenses/by/4.0/).
